# Prediction Models for Sentinel Lymph Node Metastasis in Clinically Node‐Negative Breast Cancer: Validation of Existing Nomograms, Model Development, and Ensemble Evaluation

**DOI:** 10.1002/wjs.70466

**Published:** 2026-07-02

**Authors:** Justin James, Kirti Mehta, Osamah Al‐Qershi, Emily Schembri, Michael Law, Shomik Sengupta, Christobel Saunders

**Affiliations:** ^1^ Eastern Health Melbourne Australia; ^2^ Monash University Melbourne Australia; ^3^ University of Melbourne Melbourne Australia

## Abstract

**Background:**

Axillary management in early breast cancer (EBC) is increasingly moving toward surgical de‐escalation. While sentinel lymph node biopsy (SLNB) has reduced morbidity compared with axillary lymph node dissection, it still carries risks and may be unnecessary in selected patients with very low nodal risk. An accurate estimate of the risk of sentinel lymph node (SLN) metastasis may support risk‐adapted omission strategies. This study aimed to validate existing prediction models, develop a cohort‐derived model, and evaluate whether ensemble modeling improves the prediction of SLN macrometastasis in clinically node‐negative (cN0) EBCs.

**Methods:**

We conducted a retrospective cohort study of 1080 women with cN0 EBCs treated at a tertiary center between 2012 and 2020. Existing prediction tools (Memorial Sloan Kettering Cancer Center Nomogram (MSKCCN) and MD Anderson Cancer Center Nomogram (MDACCN) were externally validated. A cohort‐derived generalized linear model (GLM) was developed using prespecified clinicopathological predictors. Two ensemble approaches combining model predictions were also evaluated. Model performance was assessed using discrimination (area under the receiver operating characteristic curve, AUC), calibration, and Brier score. Threshold‐based clinical performance was evaluated at predicted risk cut‐offs of ≤ 5% and ≥ 15%.

**Results:**

Among 1080 patients, 187 (17.3%) had nodal macrometastases. The MDACCN achieved an AUC of 0.78 (95% CI 0.74–0.82). The cohort‐derived GLM demonstrated improved discrimination with an AUC of 0.83 (95% CI 0.71–0.93) and a Brier score of 0.108. A logistic ensemble model achieved an AUC of 0.82 (95% CI 0.69–0.93) and the lowest Brier score (0.103). At *a* ≤ 5% predicted risk threshold, the GLM classified 24% of patients as low risk (observed macrometastasis rate 3.8%), while the ensemble classified 37% as low risk (observed rate 2.5%) with a false‐negative rate of 5.6%.

**Conclusions:**

Prediction models using routinely available clinicopathological variables can identify a subgroup of patients with a very low risk of SLN macrometastasis. Ensemble modeling modestly improved classification performance and identified a larger low‐risk group. Risk‐adapted prediction tools may support selective axillary de‐escalation in cN0 EBCs.

## Graphical abstract

Prediction models using routine clinicopathological variables can identify patients with a very low risk of sentinel lymph node macrometastasis. Ensemble modeling modestly improves classification and increases the proportion of patients eligible for safe, risk‐adapted axillary de‐escalation.

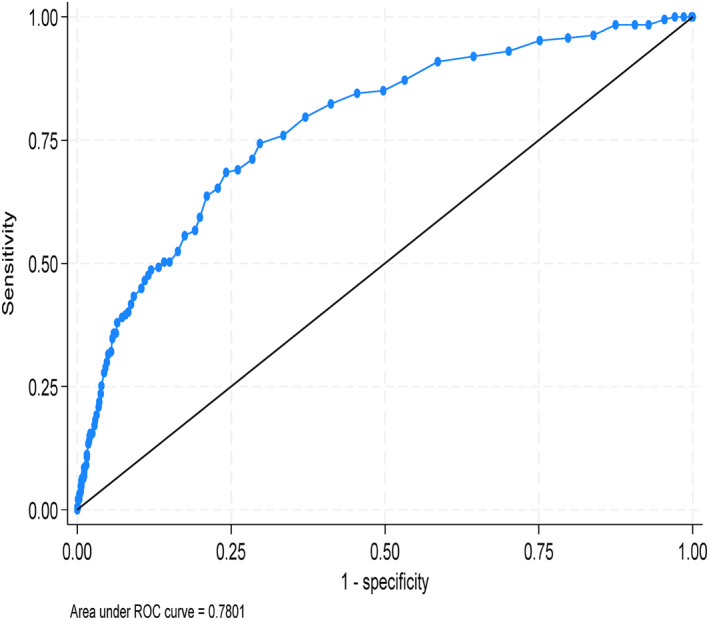

## 1 Introduction

Management of the axilla in early breast cancer (EBC) has evolved substantially over the past 2 decades. Randomised trials including ACOSOG Z0011, IBCSG 23‐01, and AMAROS demonstrated that axillary lymph node dissection (ALND) offers limited therapeutic benefit in patients with minimal or low‐volume nodal disease, enabling a shift toward de‐escalated surgery in selected patients with clinically node‐negative (cN0) EBC [1, 2, 3]. Although sentinel lymph node biopsy (SLNB) has substantially reduced the morbidity associated with axillary surgery, it is not without complications, including lymphedema, sensory disturbance, and shoulder dysfunction [4]. More recently, trials such as SOUND and INSEMA have extended axilla de‐escalation options by suggesting that SLNB itself may be safely omitted in patients with small (T1‐2) lesions and a negative axillary ultrasound [5, 6].

However, in clinical practice, the criteria for selecting patients for SLNB omission remain highly conservative. The ASCO 2025 guidelines provide a narrower clinicopathological criterion for omitting SLNB. SLNB omission is only recommended in a highly selected low‐risk subgroup—more restrictive than the trials—explicitly requiring negative axillary imaging and a treatment context where nodal status would not alter adjuvant recommendations [7]. The 2025 St Gallen International Consensus highlighted that ultrasound‐ or size‐based SLNB omission strategies such as SOUND and INSEMA may miss up to 15% of node‐positive cases, raising concerns about oncologic safety, particularly outside controlled trial settings [8].

Although SOUND and INSEMA reported excellent 5‐year survival and regional control, there are reasons to believe that short‐term non‐inferiority may not fully capture the long‐term consequences of occult nodal disease [9]. Experience from Z0011, IBCSG 23‐01, and AMAROS demonstrated that regional recurrences can continue to accrue beyond 5–10 years [10, 11, 12]. Investigators have similarly argued that follow‐up shorter than 10 years is insufficient to detect meaningful differences in regional recurrence [13, 14, 15]. In addition, pooled EBCTCG meta‐analyses have shown that even limited nodal metastasis significantly increases the risk of late distant recurrence (5–20 years), underscoring the biological and prognostic importance of accurately identifying node‐positive disease at baseline [16]. Taken together, these data emphasize that omission strategies with even modest false‐negative rates (FNR) (10%–15%) may appear safe at 5 years yet still carry long‐term oncologic uncertainty, especially for hormone receptor–positive cancers prone to late relapse.

Importantly, despite the trend toward de‐escalation, sentinel lymph node (SLN) status continues to underpin major locoregional and systemic treatment decisions in EBC. Trials such as Z0011, AMAROS, MA.20, and EORTC 22922 use SLN status to determine the need for regional nodal irradiation or additional axillary treatment [1, 3, 17, 18]. Similarly, systemic therapy decisions—including chemotherapy benefit in node‐positive postmenopausal women (RxPONDER) and genomic test interpretation (TAILORx)—are directly influenced by the presence or absence of nodal metastasis [19, 20]. EBCTCG analyses further confirm that SLN status remains one of the strongest predictors of early and late distant recurrence, highlighting its continued clinical relevance [21]. Therefore, when SLNB is omitted, accurate estimation of SLN metastasis risk remains essential to avoid inadvertent undertreatment and to ensure that radiotherapy and systemic therapy decisions reflect the true extent of disease. Precise risk estimation of SLN metastasis may be sufficient rather than accurate identification of SLN metastasis in cN0 EBC patients, given the lack of proven therapeutic benefit from SLNB in such cases.

To improve precision in patient selection, multivariable risk prediction tools such as the Memorial Sloan Kettering Cancer Center Nomogram (MSKCCN) and the MD Anderson Cancer Center Nomogram (MDACCN) were developed to estimate the probability of SLN metastasis using clinicopathological variables [22, 23]. These models have been externally validated in multiple populations, although their calibration varies across cohorts [24, 25]. Notably, no previous study has directly compared MSKCCN and MDACCN predictions within the same modern, ultrasound‐staged cN0 population, nor evaluated whether consistent low‐risk classification by both nomograms yields a safer strategy for SLNB omission. In this study, we explored whether combining models through ensemble methods yielded incremental improvements in discrimination or calibration. By comparing established nomograms with a cohort‐derived model and evaluating the incremental value of ensemble approaches, this study seeks to clarify the most reliable framework for SLN risk estimation in contemporary EBC management.

## 2 Methods

We conducted a retrospective cohort study of women with cN0 EBCs treated at a tertiary Australian center between 2012 and 2020. All patients underwent primary surgery with axillary staging by SLNB or ALND. Nodal outcome was defined as the presence of macrometastatic disease, as identified on final histopathology of SLNB and/or ALND specimens. The cohort was used to externally validate existing prediction models, develop a cohort‐derived clinical model, and evaluate ensemble modeling approaches.

We previously reported the validation of the MSKCCN [26]. The MDACCN was validated on the entire cohort using published model specifications. For each patient, predicted probabilities of SLN macrometastasis were generated using the online calculator. Model performance was assessed using discrimination, calibration, and overall accuracy metrics. Discrimination was quantified using the area under the receiver operating characteristic curve (AUC) with 95% confidence intervals (CI). Calibration was evaluated graphically using calibration plots and descriptively based on agreement between predicted and observed risk. Overall accuracy was summarized using the Brier score.

Several alternative machine‐learning approaches were explored to develop a higher‐performing model. Final model development was performed using an H2O.ai artificial intelligence framework, evaluating multiple candidate algorithms; a generalized linear model (GLM) demonstrated the best overall performance and interpretability. Candidate predictors were prespecified a priori based on a systematic review of existing mathematical prediction models and included tumor size, histological grade, lymphovascular invasion (LVI), multifocality, tumor location, age, and receptor status (ER, PR and HER2). Tumor size was modeled as a continuous variable. Age was examined as both a continuous variable and a three‐category variable (< 50, 50–70, > 70 years), and the latter was retained for its clinical interpretability and more stable model behavior. No outcome‐driven variable selection was undertaken. Three key predictors—tumor size, histological grade, and LVI—were derived from the final pathology report to define an idealized benchmark model, analogous to the development of existing nomograms, with the understanding that preoperative imaging‐based surrogates will be required for future clinical application. A cohort‐derived generalized linear model (GLM) demonstrated the best discrimination and calibration and was therefore selected for further evaluation.

The cohort was randomly divided into a development cohort (90%) and an internal validation cohort (10%), stratified by nodal outcome. A GLM with a binomial distribution and a logit link was fitted using only the development cohort, with regression coefficients estimated by maximum likelihood. Model performance was assessed in the internal validation cohort using AUC, calibration plots, calibration intercept and slope, and Brier score.

To assess whether combining predictions from multiple models could improve performance, two ensemble approaches were evaluated using predicted probabilities from the MSKCCN, MDACCN and the new GLM. The primary ensemble used a logistic combination of the three predicted probabilities, with model weights estimated solely from the development cohort. As a sensitivity analysis, a simple unweighted mean of the three predicted probabilities was also evaluated. No outcome data from the internal validation cohort were used during ensemble construction. Ensemble performance was evaluated solely in the internal validation cohort, using the same discrimination, calibration, and accuracy metrics as for the individual models.

Prespecified risk thresholds of ≤ 5% and ≥ 15% were selected a priori to explore potential low‐ and high‐risk clinical strata. Given the differing intended clinical applications of these thresholds, model performance at the ≤ 5% threshold was evaluated using the observed macrometastasis rate, negative predictive value (NPV), and FNR to prioritize safety and minimize missed nodal disease, whereas performance at the ≥ 15% threshold was assessed using the observed macrometastasis rate and positive predictive value (PPV) and sensitivity to evaluate enrichment for nodal metastasis within the higher‐risk group.

CI for AUCs and Brier scores were estimated using bootstrap resampling, whereas those for threshold‐based performance measures were calculated using exact binomial (Clopper–Pearson) methods. CI for regression coefficients and odds ratios were derived from the fitted models.

## 3 Results

A total of 1080 patients with cN0 EBCs were included in the study. Baseline demographic, tumor, and pathological characteristics of the cohort are summarized in Table 1. Overall, 187 patients (17.3%) had at least one axillary macrometastasis.

**TABLE 1:** Clinicopathological characteristics of 1080 patients in the study cohort.

Clinicopathological variables	Node negative (*n* = 893)	Node positive (*n* = 187)
Age (years), mean (SD)	61.9 (12.4)	60.0 (13.7)
Palpable lesion, *n* (%)	531 (59.5%)	152 (81.3%)
Tumor size (mm), median (IQR)	17 (11–25)	24 (18–35)
UIQ location, *n* (%)	127 (14.2%)	13 (7.0%)
Multicentricity, *n* (%)	113 (12.7%)	29 (15.5%)
LVI present, *n* (%)	78 (8.7%)	86 (46.0%)
Histological subtype and grade *n* (%):		
Grade 1 IDC	201 (22.5%)	22 (11.8%)
Grade 2 IDC	324 (36.3%)	64 (34.2%)
Grade 3 IDC	252 (28.2%)	66 (35.3%)
ILC	111 (12.4%)	35 (18.7%)
ER positive, *n* (%)	783 (87.7%)	170 (90.9%)
PR positive, *n* (%)	717 (80.3%)	160 (85.6%)
HER2 positive, *n* (%)	82 (9.2%)	26 (13.9%)
Triple negative, *n* (%)	97 (10.9%)	11 (5.9%)

*Note:* UIQ, upper inner quadrant; LVI, lymphovascular invasion; IDC, invasive ductal carcinoma; ILC, invasive lobular carcinoma; ER, estrogen receptor; PR, progesterone receptor; HER2, human epidermal growth factor receptor 2; ER positivity was defined as greater than 10% nuclear staining; SD, standard deviation; IQR, interquartile range.

**FIGURE 1:**
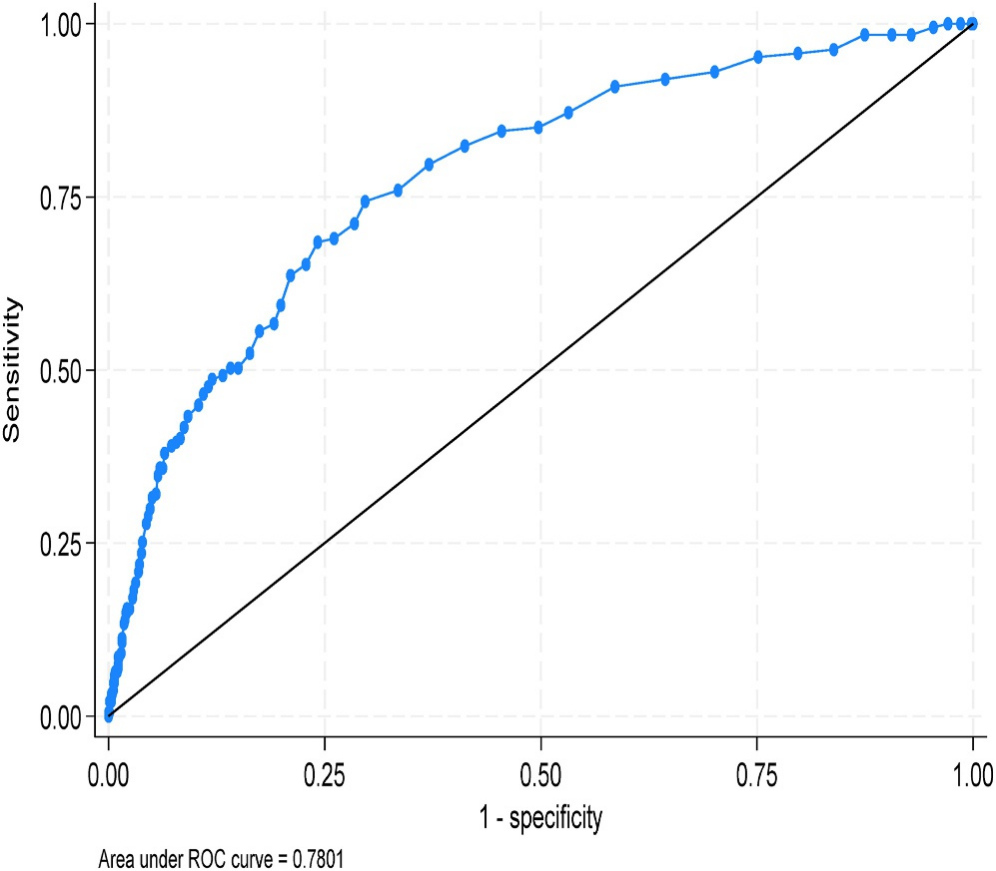
ROC curve for the MDACCN. The model demonstrated good discrimination for nodal macrometastasis on the whole cohort (*N* = 1080), with an AUC of 0.78 (95% CI, 0.74–0.82).

**FIGURE 2:**
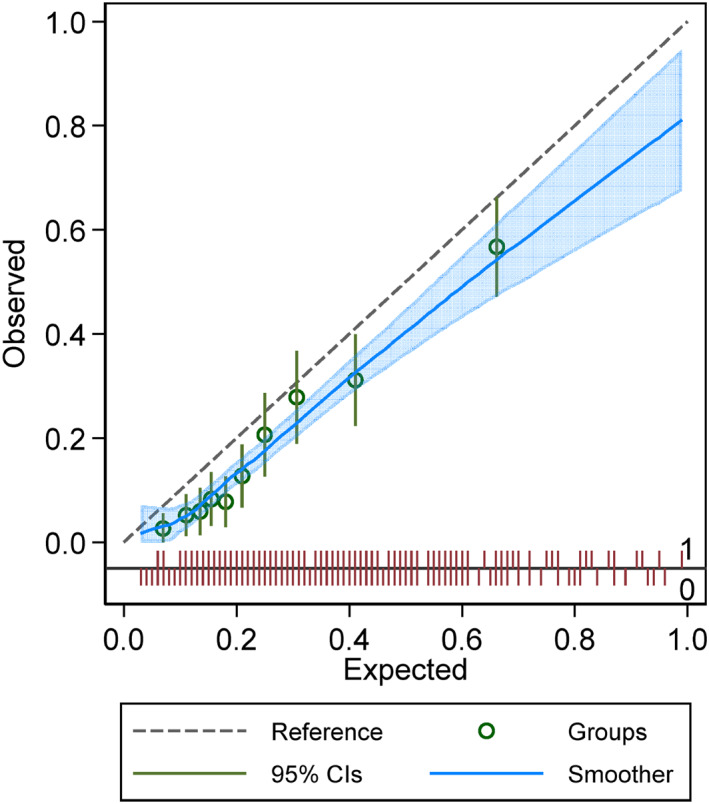
Calibration plot for the MDACCN. Calibration was assessed in the whole cohort (*N* = 1080). Observed event rates are plotted against mean predicted probabilities within deciles of risk. The dashed line represents perfect calibration. The model showed acceptable overall calibration, with a calibration intercept of −0.48 (95% CI −0.72 to −0.25) and a calibration slope of 1.04 (95% CI 0.88–1.21).

The MDACCN achieved an AUC of 0.78 (95% CI, 0.74–0.82) for predicting nodal macrometastasis (Figure 1). The calibration slope was 1.04 (95% CI 0.88–1.21), and the calibration intercept was −0.48 (95% CI −0.72 to −0.25) (Figure 2). The Brier score was 0.125 (95% CI 0.116–0.134). Model‐level performance of the MDACCN and MSKCCN (adapted from James et al.) applied to the same cohort is summarized in Table 2.

**TABLE 2:** Model‐level predictive performance of MSKCCN and MDACCN.

Performance metric	MSKCCN (James et al. [26])	MDACCN (current study)
Discrimination (AUC, 95% CI)	0.77 (0.73–0.80)	0.78 (0.74–0.82)
Overall accuracy (Brier score)	0.174	0.125 (95% CI 0.116–0.134)
Calibration (qualitative)	Tendency toward overestimation of nodal risk, most pronounced at higher predicted probabilities	Systematic overestimation of absolute nodal risk with preserved risk stratification

*Note:* Both nomograms were evaluated in the same cohort of clinically and sonographically node‐negative breast cancer patients. MSKCC performance metrics are taken from James et al. AUC, area under the receiver operating characteristic curve; CI, confidence interval; MSKCCN, Memorial Sloan Kettering Cancer Center Sentinel Node Nomogram; MDACCN, University of Texas MD Anderson Cancer Center Nomogram.

**FIGURE 3:**
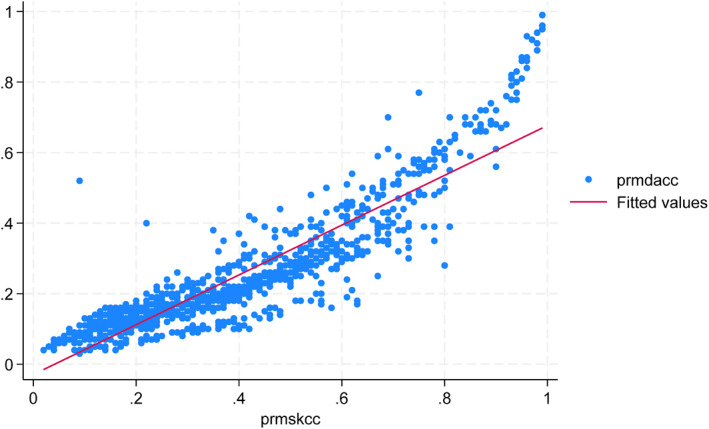
Individual‐patient agreement between MDACCN and MSKCCN predicted nodal risk. Scatter plot comparing individual‐patient predicted probabilities of nodal positivity generated by the MDACCN and MSKCCN. Variables are highly correlated (Pearson's rho = 0.905, *p* < 0.001). The diagonal line represents perfect agreement. The wide dispersion of points around the line indicates variability in risk estimates between the two models across the spectrum of predicted probabilities.

The agreement between MDACCN and MSKCCN in predicting nodal risk was assessed at the individual‐patient level. Substantial dispersion was observed in predicted probabilities between the two models (Figure 3). The median absolute difference in predicted risk was 0.056. An absolute difference of at least 5% was observed in 61.3% of patients, and an absolute difference of at least 10% in 22.7%.

**FIGURE 4:**
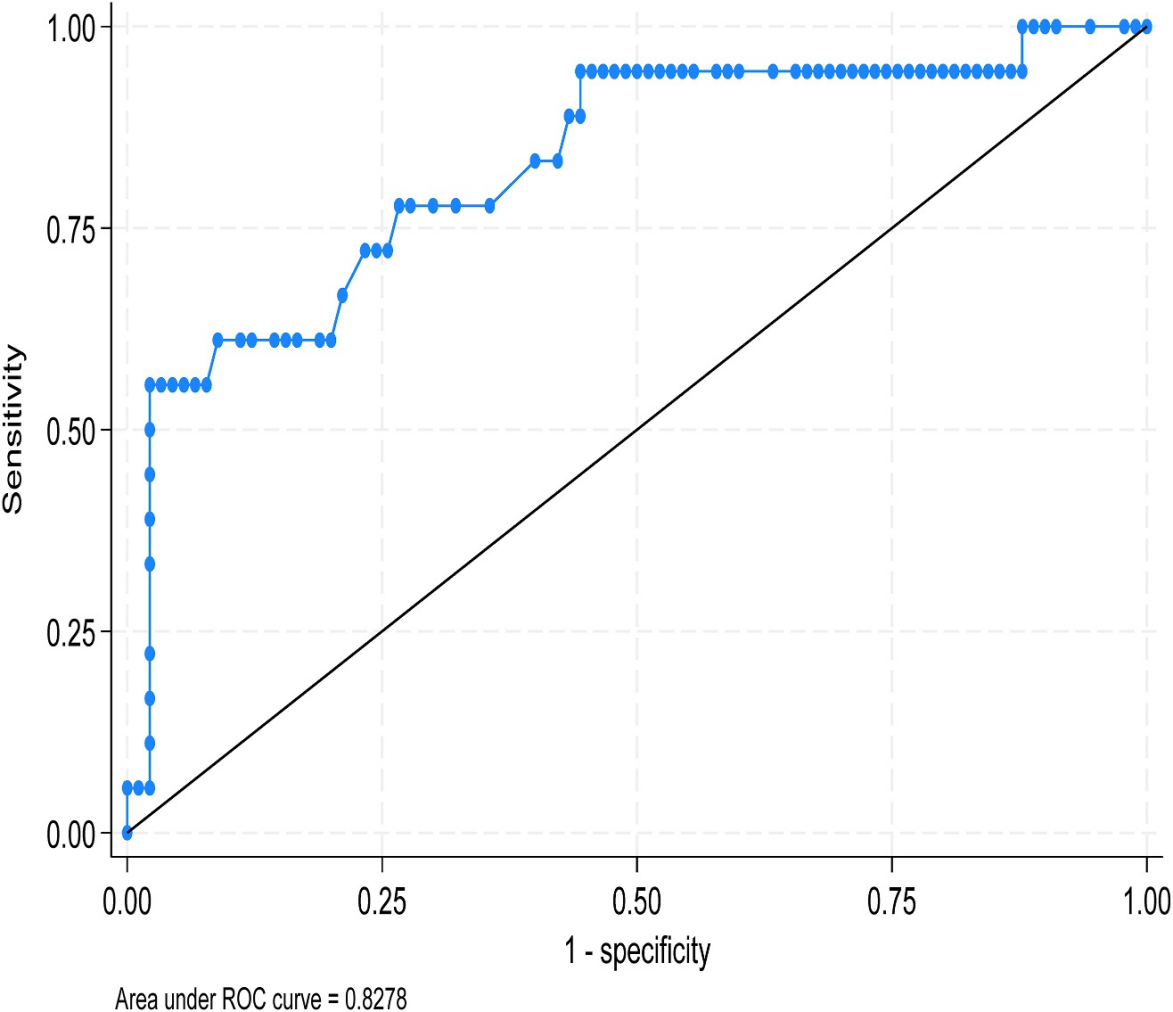
Receiver operating characteristic (ROC) curve for the cohort‐derived generalized linear model (GLM) in the internal validation cohort. The ROC curve illustrates the discriminative performance of the cohort‐derived GLM for predicting sentinel lymph node macrometastasis in the internal validation (test) cohort. Sensitivity is plotted against 1 − specificity across the full range of predicted probabilities. The dashed diagonal line represents chance‐level discrimination.

**FIGURE 5:**
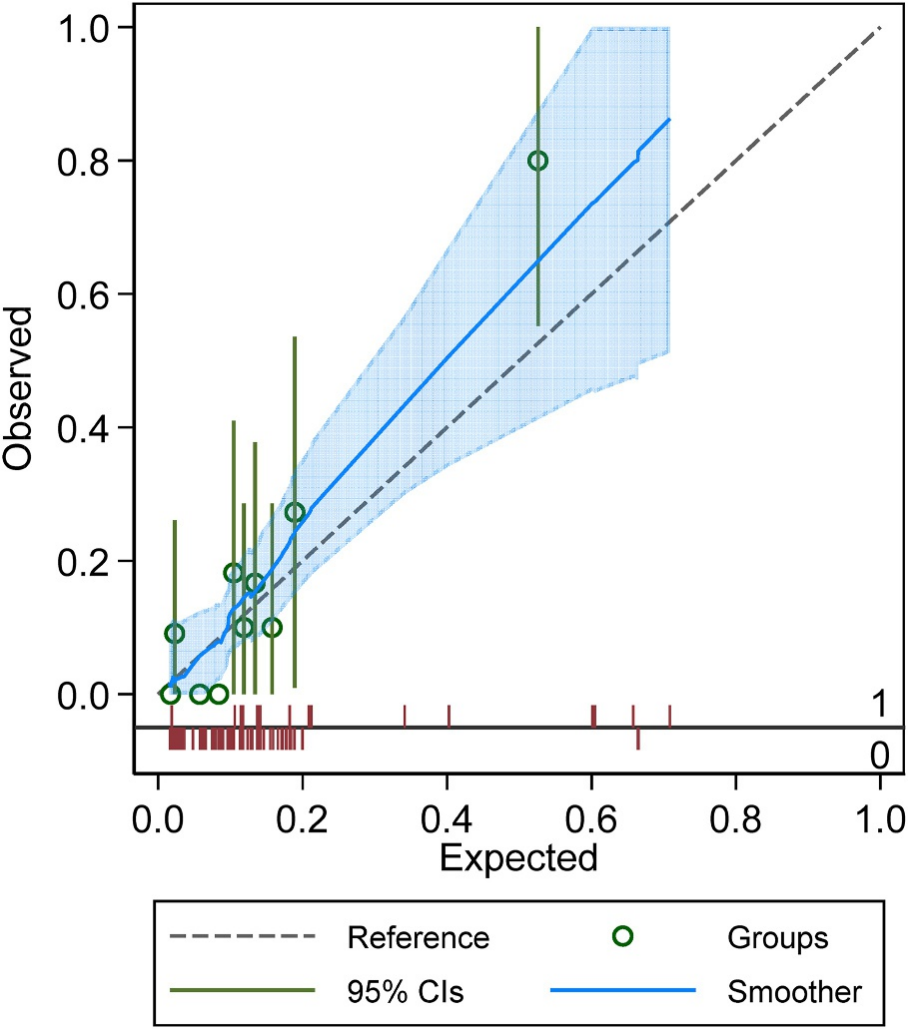
Calibration plot for the cohort‐derived generalized linear model (GLM) in the internal validation cohort. The calibration plot depicts agreement between predicted and observed risk of sentinel lymph node macrometastasis for the cohort‐derived GLM in the internal validation (test) cohort. Observed event rates are shown across deciles of predicted risk with 95% confidence intervals, together with a smoothed calibration curve. The dashed diagonal line indicates perfect calibration.

Variables selected a priori based on clinical relevance and prior nomograms were entered into multivariable modeling. Independent associations with nodal macrometastasis are shown in Table 3. Larger tumor size and LVI demonstrated the strongest independent associations with nodal macrometastasis, while tumor location in the UIQ was associated with lower odds. Histological grade and age category demonstrated weaker independent effects following adjustment for other predictors. The GLM developed from these variables achieved an AUC of 0.828 (95% CI 0.708–0.933) and a Brier score of 0.108 (Figure 4). The calibration slope was 1.22, and the calibration intercept was 0.65 (Figure 5).

**TABLE 3:** Multivariable logistic regression estimates for predictors included in the final clinical GLM.

Predictor	OR	95% CI	*p* value
Tumor size (per mm)	1.03	1.02–1.04	< 0.001
Histological grade I versus II	0.78	0.44–1.37	0.378
Histological grade III versus II	0.84	0.55–1.28	0.408
LVI (present vs absent/unknown)	7.92	5.26–11.93	< 0.001
Tumor location (UIQ vs others)	0.46	0.23–0.90	0.023
Age < 50 versus 50–70 years	1.35	0.83–2.20	0.221
Age > 70 versus 50–70 years	0.99	0.64–1.55	0.974

*Note:* Final multivariable logistic regression model for prediction of nodal macrometastasis. ORs represent the independent association between each predictor and nodal macrometastasis in the training cohort (*n* = 972). Histological grade II and age 50–70 years were used as reference categories. LVI was modeled as a binary variable (present vs absent/unknown). Tumor size was analyzed as a continuous variable.

Abbreviations: CI, confidence interval; GLM, generalized linear model; LVI, lymphovascular invasion; OR, odds ratio; UIQ, upper inner quadrant.

Two ensemble approaches were evaluated in the internal validation cohort by combining predicted probabilities from the MSKCCN, MDACCN, and the new GLM to evaluate the incremental contribution of existing nomogram‐based predictions beyond the GLM. A logistic stacked ensemble model was fitted to the development cohort (*n* = 972) using the logit‐transformed predicted probabilities from the MSKCCN and MDACCN, along with the clinical GLM. The stacked model was specified as: logit (*P*_ensemble_) = 1.451–0.217 x logit (*P*_MSKCC_)–0.070 x logit (*P*_MDACC_) + 2.006 x logit (*P*_GLM_) where logit(*p*) = ln (*p*/(1‐p)). The ensemble‐predicted probability was obtained by applying the inverse logit transformation to the linear predictor: *P*_ensemble_ = 1/(1 + exp (‐logit (*P*_ensemble_))).

**FIGURE 6:**
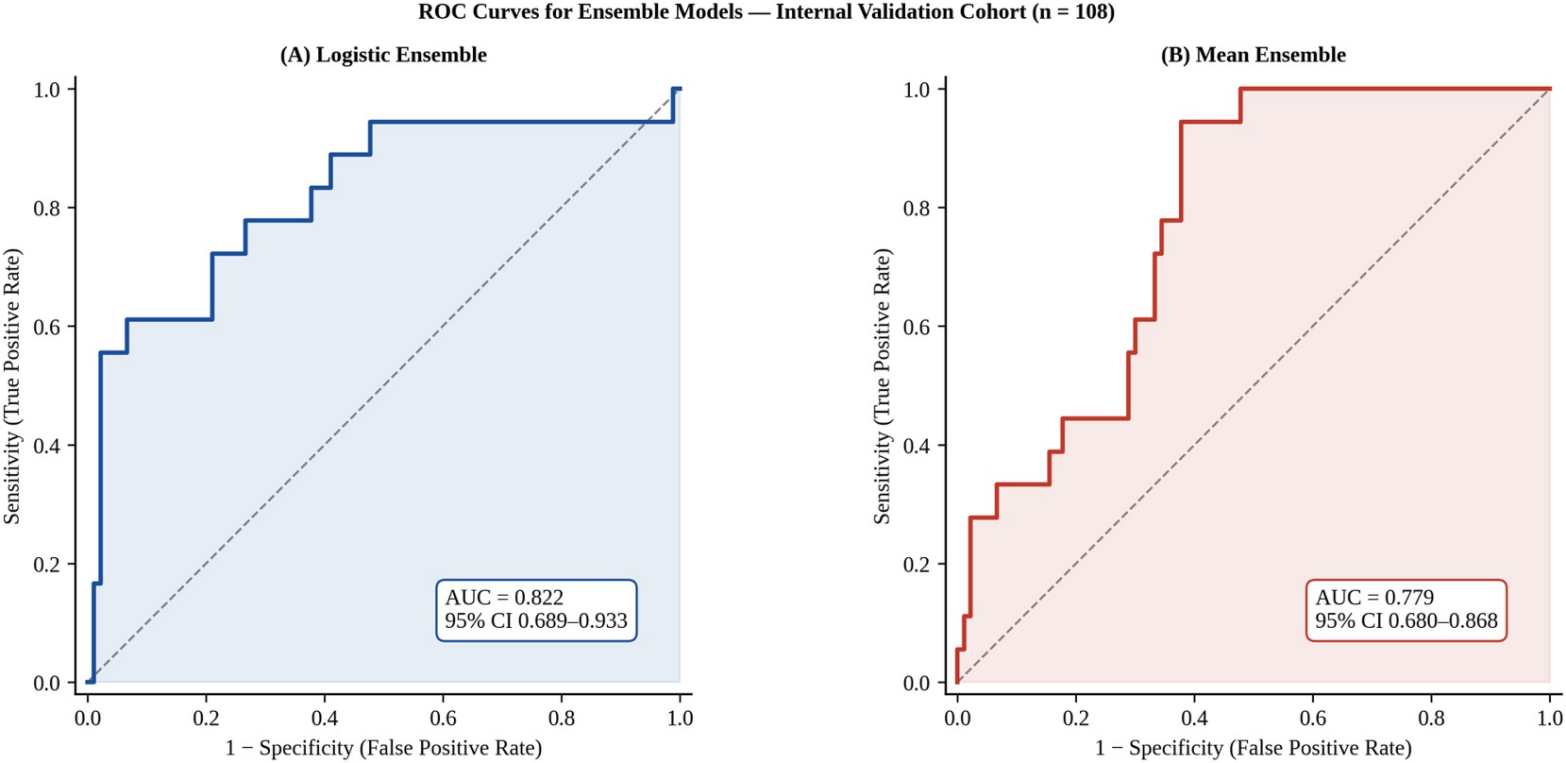
Receiver Operating Characteristic (ROC) Curves for Ensemble Models in the Internal Validation Cohort. Receiver operating characteristic (ROC) curves for the logistic ensemble (Panel A) and mean ensemble (Panel B) in the internal validation cohort (*n* = 108; 18 sentinel lymph node macrometastasis events). Each panel displays the true positive rate (sensitivity) against the false positive rate (1 − specificity) across all prediction thresholds. The dashed diagonal line represents the line of no discrimination (AUC = 0.5). The shaded region beneath each curve represents the area under the ROC curve. The logistic ensemble achieved an AUC of 0.822 (95% CI 0.689–0.933) and the mean ensemble achieved an AUC of 0.779 (95% CI 0.680–0.868). 95% confidence intervals were estimated by bootstrap resampling (2000 iterations).^1^ Acknowledgment. This figure was prepared with the assistance of Claude (Anthropic, claude‐sonnet‐4‐6, 2026). ROC curves were generated using Python (v3.10) with the matplotlib library for visualization and openpyxl for data extraction from the source spreadsheet (GLM_Ensemble_DataV4. xlsx, columns AL and AM). The area under the ROC curve (AUC) was computed using the trapezoidal method; 95% confidence intervals were estimated by bootstrap resampling (2000 iterations, seed 42) applied to the internal validation cohort (*n* = 108). The Word document was assembled using the docx JavaScript library (v8). All values were verified against the manuscript results text.

The model converged without instability and demonstrated a strong overall fit (likelihood ratio test *p* < 0.001; McFadden pseudo‐R^2^ = 0.41). In the stacked model, the GLM prediction showed a strong independent association with nodal macrometastasis (*β* = 2.01, 95% CI 1.65–2.37; OR 7.44, 95% CI 5.19–10.65; *p* < 0.001). The MSKCCN and MDACCN logit‐transformed probabilities were not independently associated with nodal macrometastasis after adjustment for the GLM prediction. Regression coefficients, confidence intervals, and odds ratios for all components of the stacked model are presented in Table 4. The logistic ensemble achieved an AUC of 0.822 (95% CI 0.689–0.933), a Brier score of 0.103 (Figure 6), a calibration slope of 0.66, and a calibration intercept of −0.22. (Supporting Information S1 Appendix 1).

**TABLE 4:** Logistic stacked ensemble model coefficients for prediction of nodal macrometastasis (development cohort).

Predictor	β (95% CI)	Adjusted OR (95% CI)	*p*‐value
Intercept	1.45 (0.76–2.14)	4.27 (2.13–8.54)	< 0.001
logit (MSKCCN probability)	−0.22 (−0.79 to 0.36)	0.80 (0.45–1.43)	0.46
logit (MDACCN probability)	−0.07 (−0.76 to 0.62)	0.93 (0.47–1.85)	0.84
logit (GLM probability)	2.01 (1.65–2.37)	7.44 (5.19–10.65)	< 0.001

*Note:* The model incorporated logit‐transformed predicted probabilities from the MSKCC and MDACC nomograms, as well as from the cohort‐derived generalized linear model (GLM). The ensemble probability was obtained by applying the inverse logit transformation to the linear predictor. Coefficients were estimated in the development cohort and applied unchanged to the validation cohort.

Abbreviations: CI, confidence interval; GLM, generalized linear model; MDACCN, University of Texas MD Anderson Cancer Center Nomogram; MSKCCN, Memorial Sloan Kettering Cancer Center Sentinel Node Nomogram; OR, odds ratio.

A simple mean ensemble was also examined by averaging the probabilities from the three models. This mean ensemble achieved an AUC of 0.779 (95% CI 0.680–0.868), a Brier score of 0.125, a calibration slope of 1.20, and a calibration intercept of −0.44. Performance metrics for the individual models and the ensemble approaches are summarized in Table 5.

**TABLE 5:** Comparative performance of individual models and ensemble approaches in the internal validation cohort.


Model	AUC (95% CI)	Brier score	Calibration slope	Calibration intercept	Qualitative calibration summary
MSKCCN	0.694 (0.558–0.814)	0.186	0.55	−1.41	Systematic overestimation; predictions too extreme
MDACCN	0.733 (0.602–0.855)	0.132	0.72	−0.92	Systematic overestimation; predictions too extreme
GLM	0.828 (0.708–0.933)	0.108	1.22	0.65	Acceptable calibration; slight underestimation
Ensemble (logistic combination)	0.822 (0.689–0.933)	0.103	0.66	−0.22	Acceptable overall calibration; predictions somewhat too extreme
Ensemble (simple mean)	0.779 (0.680–0.868)	0.125	1.20	−0.44	Systematic overestimation; predictions slightly too moderate

Abbreviations: AUC, area under the receiver operating characteristic curve; CI, confidence interval; GLM, generalized linear model; MDACCN, University of Texas MD Anderson Cancer Center Nomogram; MSKCCN, Memorial Sloan Kettering Cancer Center Sentinel Node Nomogram.

Threshold‐based performance of the cohort‐derived GLM and ensemble models was evaluated in the internal validation cohort using prespecified risk cut‐offs of 5% and 15% (Table 6). At *a* ≤ 5% predicted risk threshold, the GLM classified 26 patients (24.1%) as low risk, among whom the observed macrometastasis rate was 3.8% (95% CI 0.1–19.6), with a negative predictive value (NPV) of 96.2% (95% CI 80.4–99.9) and a false‐negative rate (FNR)of 5.6% (95% CI 0.1–27.3). The logistic ensemble classified 40 patients (37.0%) as low risk at the same threshold, with an observed macrometastasis rate of 2.5% (95% CI 0.1–13.2), a NPV of 97.5% (95% CI 86.8–99.9), and a FNR of 5.6% (95% CI 0.0–20.0). The mean ensemble classified 3 patients (2.8%) as low risk, with no observed macrometastases.

**TABLE 6:** Threshold‐based performance of the cohort‐derived GLM and ensemble models in the internal validation cohort.

Model	Threshold	Patients classified (*n*, %)	Observed macrometastasis rate (95% CI)	NPV (95% CI)	FNR (95% CI)
Cohort‐derived GLM	≤ 5%	26 (24.1%)	3.8% (0.1–19.6)	96.2% (80.4–99.9)	5.6% (0.1–27.3)
Logistic ensemble	≤ 5%	40 (37.0%)	2.5% (0.1–13.2)	97.5% (86.8–99.9)	5.6% (0.1–27.3)
Mean ensemble	≤ 5%	3 (2.8%)	0% (0–70.8)	100% (29.2–100)	0% (0–18.5)

Model	Threshold	Patients classified (*n*, %)	Observed macrometastasis rate (95% CI)	PPV (95% CI)	Sensitivity (95% CI)
Cohort‐derived GLM	≥ 15%	28 (25.9%)	39.3% (21.5–59.4)		
Logistic ensemble	≥ 15%	26 (24.1%)	42.3% (23.4–63.1)	4–61.3	61.11% (38–80.4)
Mean ensemble	≥ 15%	80 (74.1%)	22.5% (13.9–33.2)	8	

Abbreviations: AUC, area under the receiver operating characteristic curve; CI, confidence interval; Sensitivity, proportion of all true positives classified as high risk; FNR, false‐negative rate; GLM, generalized linear model; NPV, negative predictive value; PPV, positive predictive value.

At *a* ≥ 15% predicted risk threshold, the GLM classified 28 patients (25.9%) as high risk, with an observed macrometastasis rate of 39.3% (95% CI 21.5–59.4) (PPV 39.3% (95% CI 21.5–59.4)) and a sensitivity of 61.1% (95% CI 35.7–82.7). The logistic ensemble classified 26 patients (24.1%) as high risk, with an observed macrometastasis rate of 42.3% (95% CI 23.4–63.1) (PPV 42.3% (95% CI 23.4–63.1)) and a sensitivity of 61.1% (95% CI 35.7–82.7). The mean ensemble classified 80 patients (74.1%) as high risk, with an observed macrometastasis rate of 22.5% (95% CI 13.9–33.2), a PPV of 22.5% (95% CI 13.9–33.2), and a sensitivity of 100% (95% CI 81.5–100.0).

## 4 Discussion

Risk‐adapted de‐escalation using prediction models to identify patients with a very low likelihood of SLN metastasis may offer a pragmatic middle path between routine axillary surgery and universal omission. The ≤ 5% and ≥ 15% thresholds were selected a priori as clinically interpretable exploratory cut‐offs to identify potential low‐ and high‐risk groups, rather than serving as definitive treatment thresholds. The ≤ 5% threshold reflected a conservative rule‐out strategy for potential SLNB omission, recognizing the accepted FNR of approximately 8% associated with SLNB. Conversely, the ≥ 15% threshold was selected to identify patients with a higher prevalence of nodal macrometastasis, informed by the rates of occult nodal disease reported in contemporary trials investigating SLNB omission.

At the ≤ 5% threshold, the cohort‐derived GLM identified approximately one quarter of patients as low risk with an observed macrometastasis rate of 3.8%, while the logistic ensemble classified a larger proportion of patients as low risk while maintaining a similarly low FNR. At the ≥ 15% threshold, the models identified subgroups with substantially higher observed rates of nodal macrometastasis, supporting their potential role in risk stratification and patient counseling.

However, these thresholds should be interpreted cautiously, as clinically appropriate cut‐offs are likely to vary across populations and depend on local disease prevalence, imaging practices, and tolerance for missed nodal disease. External validation and local calibration would therefore be essential before any model‐derived threshold could be considered for routine clinical implementation.

Our findings also suggest a potential role for ensemble modeling approaches in clinical prediction. Although the logistic ensemble did not meaningfully improve overall discrimination compared with the cohort‐derived GLM, it demonstrated favorable threshold‐based stratification performance by identifying a larger low‐risk group at the ≤ 5% threshold while maintaining a comparable FNR. This suggests that ensemble approaches may improve clinically actionable risk stratification even when gains in global performance metrics such as AUC are modest. The incremental benefit of more complex ensemble approaches should therefore be weighed against increased model complexity and the need for additional external validation before clinical application.

The present study should also be interpreted in the context of several limitations. First, the model development and validation were performed within a relatively modest dataset, which may limit the stability of coefficient estimates and the precision of performance metrics. Although internal validation was undertaken, external validation in independent cohorts is essential before the model can be recommended for routine clinical use. Second, the event rate of SLN macrometastasis was relatively low, which may affect the precision of estimates at specific decision thresholds. Third, key predictors like LVI and histological grade were derived from postoperative pathology and are therefore not fully available at the time of preoperative decision‐making. As such, the reported model performance represents an idealized benchmark of the discriminatory potential achievable using clinicopathological variables, rather than a directly deployable preoperative tool in its current form. Nevertheless, these variables were intentionally retained because they are established predictors within existing nomograms and provide a useful framework for future development of clinically applicable surrogate measures, including biopsy‐derived pathology estimates and imaging‐ or radiomics‐based predictors. Finally, as with all retrospective modeling studies, unmeasured confounders and variations in imaging or pathological assessment may influence model performance.

Despite these limitations, the study has several strengths. The models were developed using routinely available clinicopathological variables, allowing straightforward integration into clinical workflows. In addition, evaluating clinically meaningful risk thresholds provides practical insight into how the models might support decision‐making in real‐world settings. Importantly, the framework developed here also allows incorporation of additional predictors, such as imaging‐derived radiomic features, which may further improve predictive accuracy and allow predictions without relying on postoperative histological variables.

Future work should focus on prospective validation of the model in independent cohorts and on evaluating its performance across diverse clinical settings. The integration of radiomic features to replace post‐operative histological predictors, to make the model a pure pre‐operative prediction model that allows individualized risk estimation, is a priority. Ultimately, implementation of a validated prediction model through a clinician‐accessible web interface may facilitate shared decision‐making regarding axillary surgery in patients with early breast cancer.

In conclusion, the cohort‐derived GLM and logistic ensemble models demonstrated promising performance for predicting sentinel lymph node macrometastasis in cN0 EBCs. Threshold‐based analysis suggests that these models may identify a subset of patients with a very low risk of nodal disease who could potentially avoid axillary surgery. Prospective validation and integration into clinical decision tools will be important next steps to determine the clinical utility of this approach in the evolving era of axillary de‐escalation.

## Author Contributions

**Justin James:** writing – original draft, formal analysis, data curation. **Kirti Mehta:** conceptualization, supervision. **Osamah Al‐Qershi:** methodology, formal analysis. **Emily Schembri:** formal analysis, visualization, methodology, software. **Michael Law:** conceptualization, resources, supervision. **Shomik Sengupta:** conceptualization, supervision. **Christobel Saunders:** supervision, writing – review and editing.

## Funding

This work was supported by the Eastern Health Foundation through the Linda Williams Memorial Oncology Research and Innovation Grant, awarded in two rounds (00041EHFRIG2021 and 00053EHFRIG2022). The funder had no role in study design, data analysis, interpretation, or the decision to submit the work for publication.

## Ethics Statement

This retrospective study was approved by the Eastern Health Human Research Ethics Committee (reference LR21‐020‐74578; approved 2 August 2021, amended 28 February 2022) and was conducted in accordance with the National Statement on Ethical Conduct in Human Research (2007, including all updates).

## Consent

As a low‐risk, non‐interventional study, the requirement for individual informed consent was waived by the Eastern Health Human Research Ethics Committee.

## Conflicts of Interest

The authors declare that they have no competing interests. No author is a member of the Breast Surgery International (BSI) society.

## Supporting information


Supporting Information S1

## Data Availability

The data that support the findings of this study are available from the corresponding author upon reasonable request.
