# Supplementary material for: Prediction Models for Sentinel Lymph Node Metastasis in Clinically Node‐Negative Breast Cancer: Validation of Existing Nomograms, Model Development, and Ensemble Evaluation
Source: World J Surg. 2026 Jul 2;50(8):2219–28. doi: 10.1002/wjs.70466 (PMC13460711; doi:10.1002/wjs.70466)
Supplement: Supplementary file 1 [file WJS-50-2219-s001.docx]

**Appendix 1**

**Calibration Plots for All Five Prediction Models**

*Internal Validation Cohort · n = 108 · 18 nodal macrometastases (16.7%)*

**Figure Legend.** Calibration plots for the five prediction models evaluated in the internal validation cohort. (A) Memorial Sloan Kettering Cancer Center Nomogram (MSKCCN). (B) MD Anderson Cancer Center Nomogram (MDACCN). (C) Cohort-derived generalised linear model (GLM). (D) Logistic stacked ensemble. (E) Simple mean ensemble. Each panel shows observed event rates (filled circles) within deciles of predicted risk with 95% Wilson confidence intervals, a kernel-smoothed calibration curve (solid line), the line of perfect calibration (dashed diagonal), and the distribution of predicted probabilities (rug plot along the x-axis). AUC, area under the receiver operating characteristic curve; Brier, Brier score; Int., calibration intercept; Slope, calibration slope.^[1]^

1. **Acknowledgement.** This appendix was prepared with the assistance of Claude (Anthropic, claude-sonnet-4-6, 2026). Calibration plots were generated using Python (v3.10) with the matplotlib library (v3.10) for visualisation and openpyxl for data extraction from the source spreadsheet. Statistical calculations — including calibration slope and intercept (via Newton–Raphson logistic regression), AUC (trapezoidal method), and Brier score — were computed directly from the internal validation cohort (n = 108) using columns AH, AJ, AK, AL, and AM of GLM_Ensemble_DataV4.xlsx. Calibration curves were produced using kernel-weighted smoothing (Gaussian kernel, bandwidth = 0.45 × SD of predicted probabilities). Decile grouping with 95% Wilson confidence intervals was applied to observed event rates. The Word document was assembled using the docx JavaScript library (v8). All computations were verified against the manuscript results text.
